# Identification and analysis of RNA-5-methylcytosine-related key genes in osteoarthritis

**DOI:** 10.1186/s12864-023-09651-4

**Published:** 2023-09-12

**Authors:** Yang Yu, Shitao Lu, Xiaoming Liu, Yu Li, Jianzhong Xu

**Affiliations:** 1 Department of Orthopedics, the First Affiliated Hospital of Zhengzhou University, Zhengzhou, China; 2 Department of Gastroenterology, the Third Xiangya Hospital, Central South University, Changsha, China

**Keywords:** Osteoarthritis, m5C methylation, Collagen degradation, Methylome profile, MeRIP-seq

## Abstract

**Background:**

5-methylcytosine (m5C) modification is widely associated with many biological and pathological processes. However, knowledge of m5C modification in osteoarthritis (OA) remains lacking. Thus, our study aimed to identify common m5C features in OA.

**Results:**

In the present study, we identified 1395 differentially methylated genes (DMGs) and 1673 differentially expressed genes (DEGs) using methylated RNA immunoprecipitation next-generation sequencing (MeRIP-seq) and RNA-sequencing. A co-expression analysis of DMGs and DEGs showed that the expression of 133 genes was significantly affected by m5C methylation. A protein–protein interaction network of the 133 genes was constructed using the STRING database, and the cytoHubba plug-in of Cytoscape was used to hub genes were screen out 11 hub genes, including MMP14, VTN, COL15A1, COL6A2, SPARC, COL5A1, COL6A3, COL6A1, COL8A2, ADAMTS2 and COL7A1. The Pathway enrichment analysis by the ClueGO and CluePedia plugins in Cytoscape showed that the hub genes were significantly enriched in collagen degradation and extracellular matrix degradation.

**Conclusions:**

Our study indicated that m5C modification might play an important role in OA pathogenesis, and the present study provides worthwhile insight into identifying m5C-related therapeutic targets in OA.

**Supplementary Information:**

The online version contains supplementary material available at 10.1186/s12864-023-09651-4.

## Introduction

Osteoarthritis (OA) is the most common type of arthritis and one of the leading causes of pain and disability worldwide. It is estimated that approximately 27–31 million people in the US have symptomatic OA [1]. The economic costs related to OA are predicted to be between 1% and 2.5% of the gross domestic product (GDP) of developed countries [2]. Despite marked progress in OA treatment, there is currently no effective disease-modifying therapy available to relieve the pain and inhibit the progression of OA other than joint arthroplasty. This is largely due to the incomplete understanding of the pathogenesis of OA and the deficiency of effective therapeutic targets. Thus, the further elucidation of the underlying molecular mechanism of OA is urgently needed for the development of novel therapies.

Epigenetic modification plays critical regulatory roles in various biological processes of eukaryotic cells. Hundreds of modifications have been discovered in RNA, and among these abundant modifications, 5-methylcytosine (m5C) methylation has received great attention. The m5C modification participates in multiple biological processes. It was found that ALYREF promoted mRNA export in an m5C-dependent manner, and the knockdown of NSUN2, an m5C methyltransferase, significantly reduced the cytoplasmic-to-nuclear ratios of mRNAs [3]. Yang et al. demonstrated that RNA m5C modification regulated mRNA stabilization during the maternal-to-zygotic transition in zebrafish [4]. Chen et al. found that DNA damage could induce m5C modification in mRNAs at sites of DNA damage, and this is an important mechanism of DNA repair [5]. Aberrant mRNA m5C modifications are also widely involved in the pathogenesis of a variety of diseases. The m5C modification level significantly affected the proliferation and migration of HEK293 cells [6]. Chen et al. demonstrated that m5C was significantly hypermethylated in bladder cancer and primarily enriched in oncogenic pathways. The m5C modification participated in bladder cancer pathogenesis by regulating mRNA stability [7]. A study by Luo et al. showed that decreased m5C methylation (caused by the inhibition of NSUN2) can reverse atherosclerosis and endothelial inflammation after aortic transplantation [8]. Similar to N6-methyladenosine (m6A), which is the most prevalent internal mRNA modification, m5C methylation is dynamically regulated by methyltransferase or “writers” (which catalyze the deposition of m5C), demethylase or “erasers” (which catalyze the removal of m5C), and binding protein or “readers” (which recognize the modified nucleotides). While the list of m5C readers and erasers is still under debate, the m5C writers have been acknowledged and mainly include the NOL1/NOP2/sun (NSUN) family and DNA methyltransferase 2 (DNMT2) [9]. Studies have indicated that the m5C regulators modulate the expression of a variety of oncogenes in an m5C-dependent manner [10]. An illustration of the regulatory role of m5C modification in the pathogenesis of multiple diseases might facilitate progressions in treatment. A study by Lyko et al. showed that the reduced m5C modification of tRNA by DNMT2 inhibition suppressed metabolic activity in the human cancer cell line [11]. NSUN2 promoted gastric cancer development was promoted by CDKN1C repression, and the knockdown of NSUN2 suppressed tumor growth [12].

The study of the functions of m5C modification in diverse pathological processes is gaining increasing attention and has significant implications. However, the regulatory role of m5C modification in OA pathogenesis remains unknown. With advances in high-throughput technologies, researchers have successfully used m5C-RNA immunoprecipitation (IP) to characterize m5C methylation sites in several types of cancers, such as hepatocellular carcinoma [13] and ovarian cancer [14], thereby laying a foundation for the further elucidation of the mechanism. In the present study, we collected three OA knee cartilage tissues and three normal knee cartilage tissues to obtain the first transcriptome-mRNA m5C modification profile by methylated RNA immunoprecipitation next-generation sequencing (MeRIP-seq). Significant differences in the numbers and distributions of m5C peaks were noted in OA and normal cartilage tissues. Differentially methylated genes (DMGs) were identified, and gene ontology (GO) and Kyoto Encyclopedia of Genes and Genomes (KEGG) pathway enrichment analyses were performed. We identified 11 m5C-modified hub genes via bioinformatics analyses. The pathway enrichment analysis of hub genes was performed using the ClueGO and CluePedia plugins in Cytoscape.

## Results

### Flow chart

**Fig. 1:**
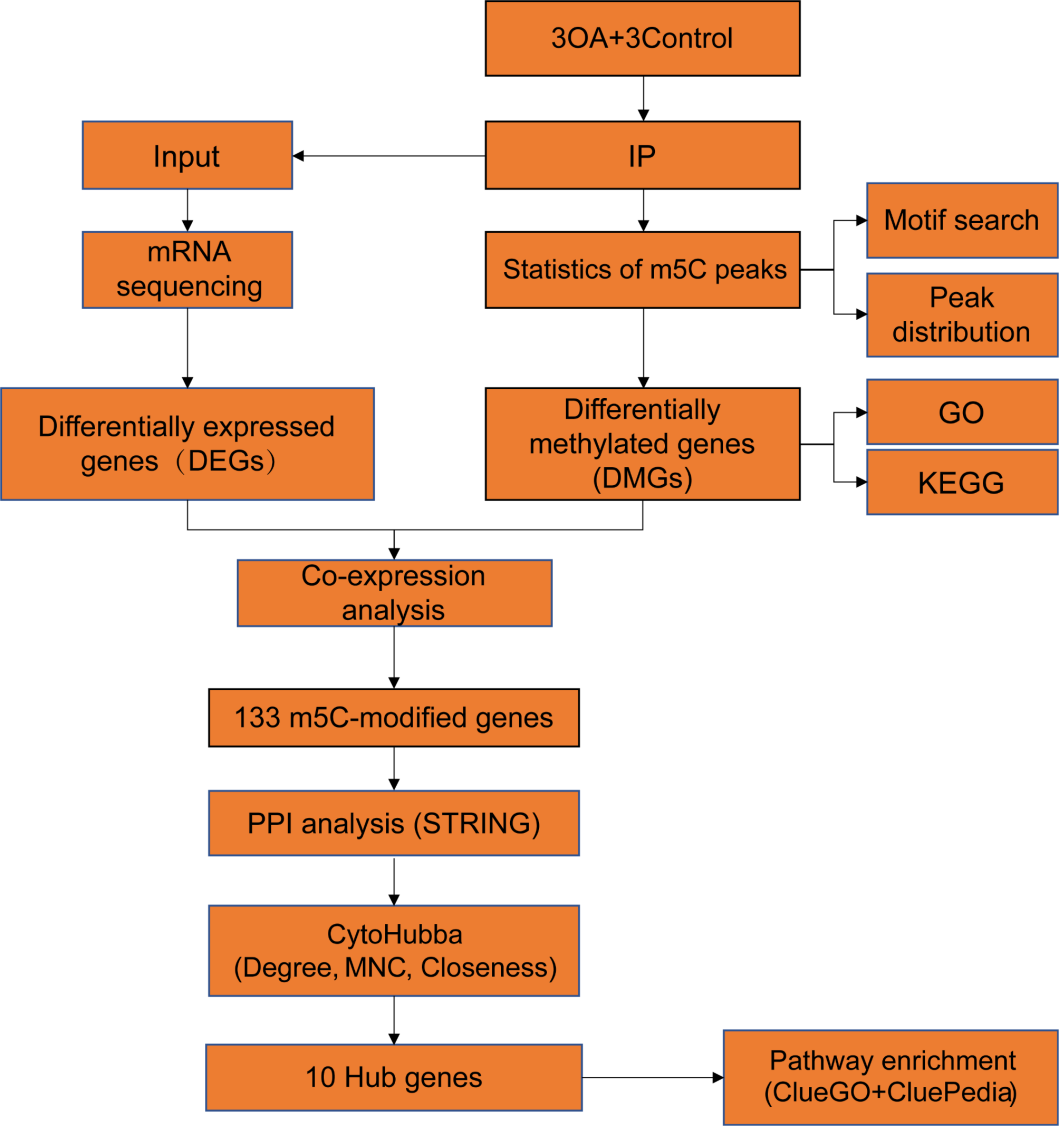
Flow chart of identification and analysis of m5C-related hub genes in OA

A flow chart of our study is shown in Fig. 1.

### General features of m5C methylation map in OA and normal knee cartilage

**Fig. 2:**
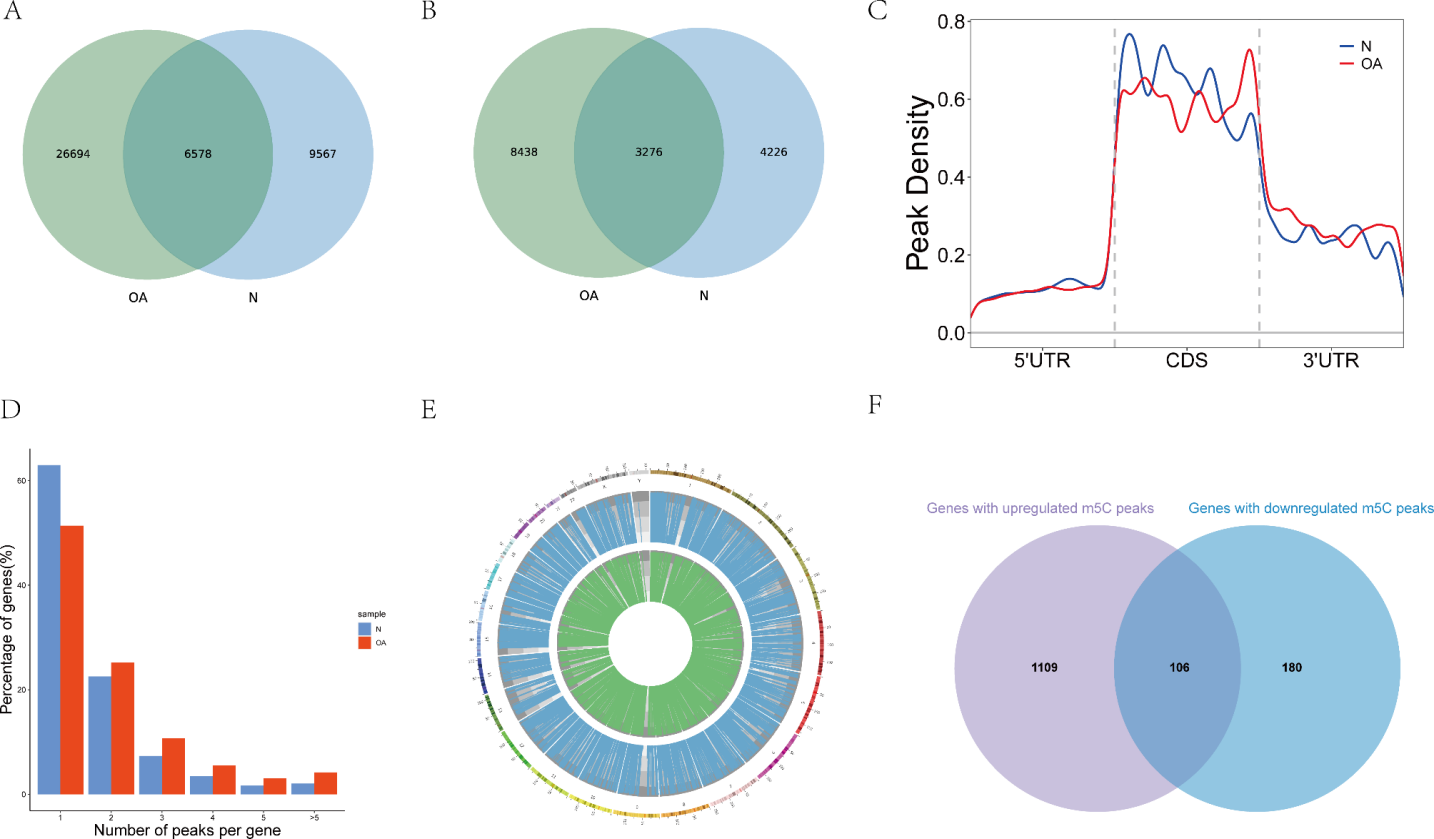
Overview of m5C peaks in mRNAs and differential m5C methylation analysis. **A** Number of m5C peaks in OA and normal groups. **B** Number of m5C methylated genes in OA and normal groups. **C** Preferential location of m5C in mRNA transcripts. Each transcript was divided into three parts: 5’untranslated region, coding DNA sequence, and 3’untranslated region. **D** The number of m5C peaks on each mRNA. Most mRNAs had only one m5C peak in both OA and normal samples. **E** Visualization of m5C at the chromosome level in OA and normal samples. **F** Number of genes with hyper- and hypomethylated m5C sites in OA cartilage

In general, we found 33,272 m5C peaks and mapped up to 11,714 annotated genes in OA samples (Fig. 2A), and we found 16,145 m5C peaks and mapped up to 7502 annotated genes in normal samples (Fig. 2B); Peak information for each sample has been listed in Additional file 1: Table S1.

To identify the preferential location of m5C in the mRNA, we analyzed the metagene profiles of the m5C peaks in the mRNA transcriptome. The m5C peaks were distributed in all regions of the mRNA, but mostly in the CDS region. The distribution in OA samples was similar to that in the normal samples at the 5′UTR and 3′UTR. The m5C peaks in OA samples were more distributed near the stop codon, and the m5C peaks in the normal samples were more distributed near the start codon (Fig. 2C). We also conducted statistical analyses of the number of peaks on each methylated mRNA. When the number of peaks detected for the same gene in different samples was inconsistent, we selected the maximum count for analysis. In the OA group, the number of genes with one, two, three, four, five, and more than five peaks are 7263, 2577, 703, 351, 176, and 234, respectively. In the normal group, the number of genes with one, two, three, four, five, and more than five peaks are 4014, 1913, 600, 375, 225, and 338, respectively. We found that most mRNAs in the OA and normal samples had only one m5C peak (Fig. 2D). We used Circos software to determine the distribution of m5C at the chromosome level. It was found that the numbers and distributions of m5C peaks on each chromosome were different between the OA and normal knee cartilage tissues (Fig. 2E).

We used DiffReps software to identify 1607 upregulated m5C methylation peaks in 1215 mRNAs and 326 downregulated m5C methylation peaks in 286 mRNAs. Among these genes, 1109 genes and 180 genes had only upregulated m5C peaks or downregulated m5C peaks, respectively, and a total of 106 genes showed both up- and downregulated m5C peaks (Fig. 2F, Additional file 2: Table S2).

### GO and KEGG pathway analysis

**Fig. 3:**
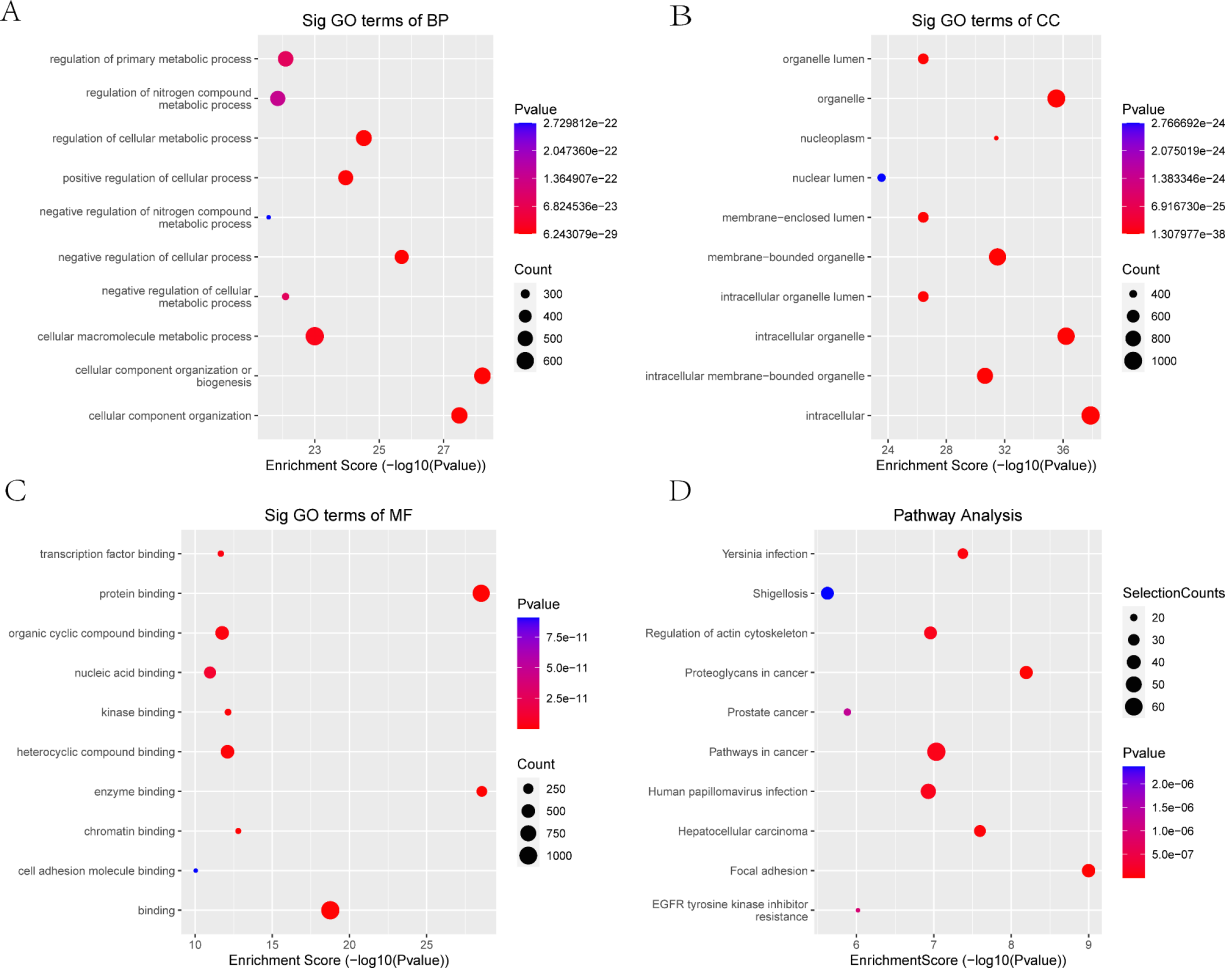
GO functional enrichment analysis and KEGG pathway analysis of mRNAs with upregulated m5C modification. **A** Biological process annotation diagram. **B** Cellular component annotation diagram. **C** Molecular function annotation diagram. **D** KEGG annotation

GO analysis indicated that the mRNAs with upregulated m5C peaks in OA cartilage were mainly enriched in the cellular component organization or biogenesis (GO term: BP) (Fig. 3A), intracellular and intracellular organelle (GO term: CC) (Fig. 3B), and protein binding and enzyme binding (GO term: MF) (Fig. 3C) categories. The KEGG analysis results showed that these mRNAs were primarily involved in focal adhesion and proteoglycans in cancer (Fig. 3D).

**Fig. 4:**
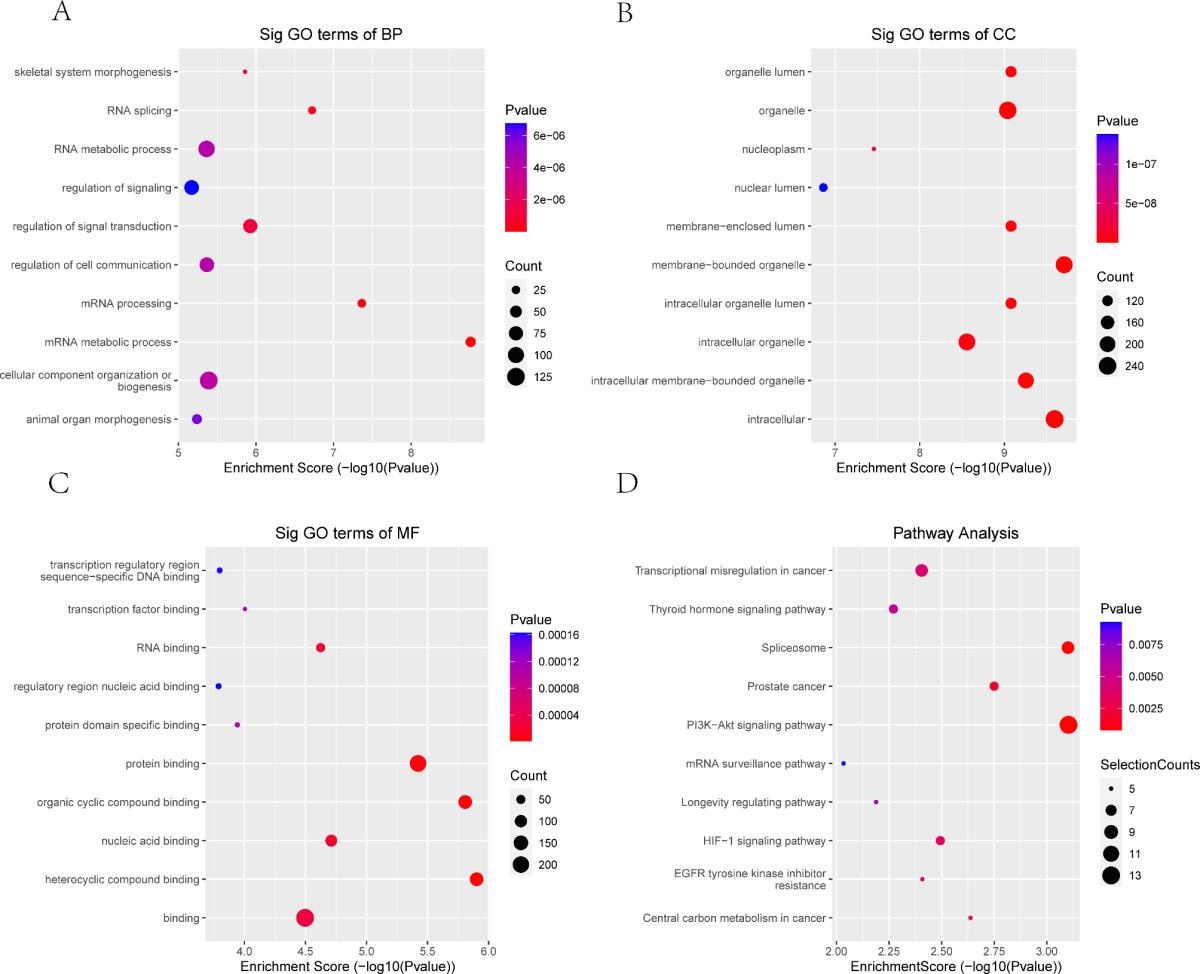
GO functional enrichment analysis and KEGG pathway analysis of mRNAs with downregulated m5C modification. **A** Biological process annotation diagram. **B** Cellular component annotation diagram. **C** Molecular function annotation diagram. **D** KEGG annotation

GO analysis indicated that the mRNAs with downregulated m5C peaks were mainly enriched in the mRNA metabolic process and mRNA processing (GO term: BP) (Fig. 4A), membrane-bounded organelle and intracellular (GO term: CC) (Fig. 4B), and heterocyclic compound binding and organic cyclic compound binding (GO term: MF) (Fig. 4C) categories. The KEGG analysis results showed that these mRNAs were significantly enriched in the PI3K-Akt signaling pathway and spliceosome (Fig. 4D).

### Association analysis of MeRIP-seq and RNA-seq data

**Fig. 5:**
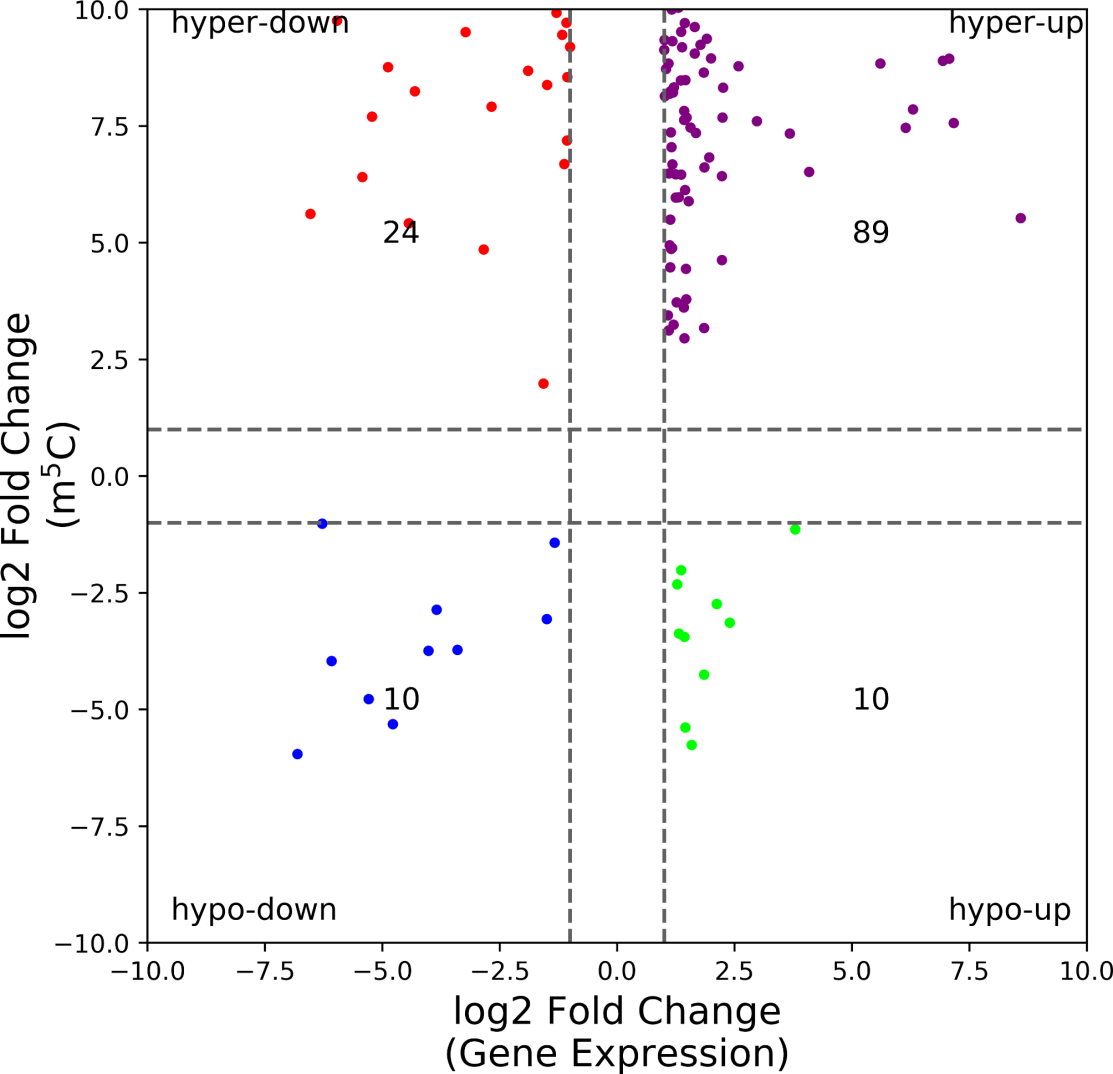
The association analysis between DEGs and DMGs. 133 genes were identified, including 89 hypermethylated and upregulated genes, 24 hypermethylated and downregulated genes, 10 hypomethylated and upregulated genes, and 10 hypomethylated and downregulated genes

In total, 1673 differentially expressed genes (DEGs) were identified in the RNA-Seq data (Additional file 3: Table S3). To explore the effect of m5C methylation on transcriptional expression in OA, we conducted a conjoint analysis of MeRIP-Seq data and RNA-Seq data. The expressions of 133 genes were affected by m5C methylation, i.e., 89 hypermethylated and upregulated genes, 24 hypermethylated and downregulated genes, 10 hypomethylated and upregulated genes, and 10 hypomethylated and downregulated genes (Fig. 5, Additional file 4: Table S4).

### Identification and the pathway enrichment analysis of hub genes

**Fig. 6:**
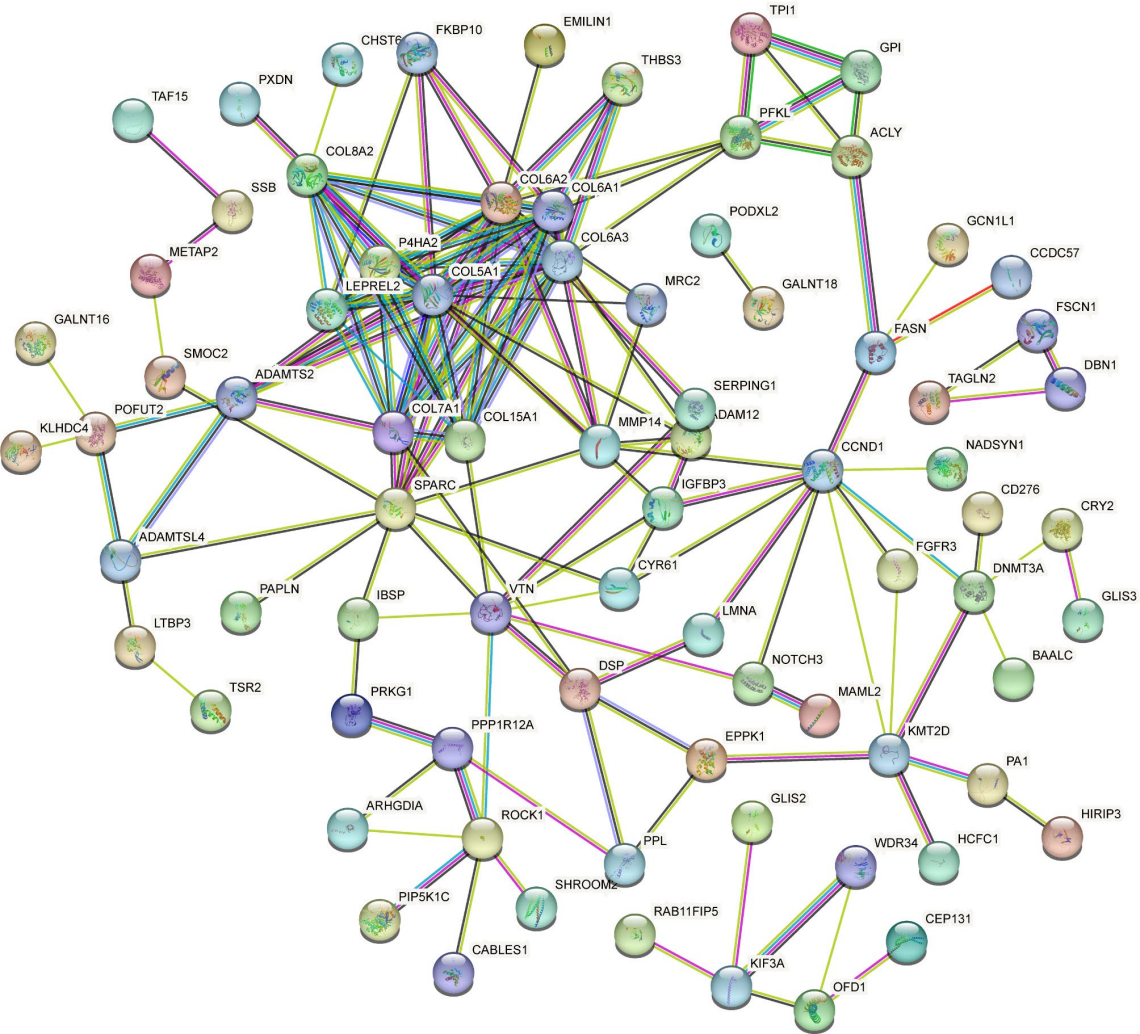
A protein–protein interaction network of the 133 genes constructed using the STRING database

**Fig. 7:**
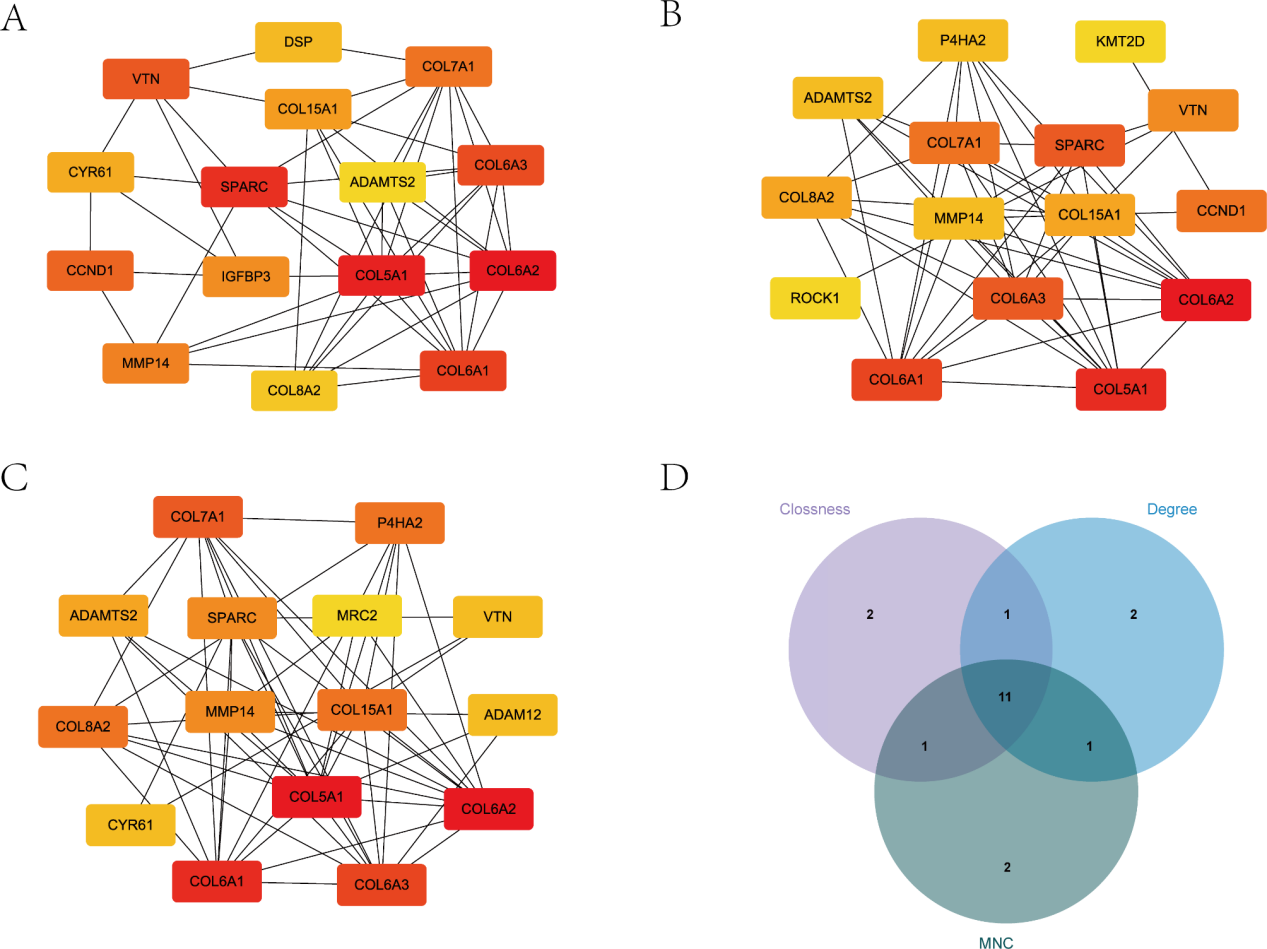
Identification of the m5C-modified hub genes in OA. **A** Top 15 genes ranked by Closeness algorithm. **B** Top 15 genes ranked by Degree algorithm. **C** Top 15 genes ranked by MNC algorithm. **D** Venn diagram of the identified genes by the three algorithms

The STRING database was used to construct a PPI network of the 133 selected genes (Fig. 6). We used the cytoHubba plug-in of Cytoscape to further analyze the PPI network. Node scores were calculated using the three algorithms of cytoHubba, and the top 15 genes were selected as key genes (Fig. 7A-C). Taking the intersections of the key genes obtained by these algorithms, 11 genes, i.e., MMP14, VTN, COL15A1, COL6A2, SPARC, COL5A1, COL6A3, COL6A1, COL8A2, ADAMTS2 and COL7A1, were identified as hub genes (Fig. 7D). All 11 genes were upregulated and hypermethylated in OA cartilage. The m5C-modified sites of these genes were shown in Table 1.

**Fig. 8:**
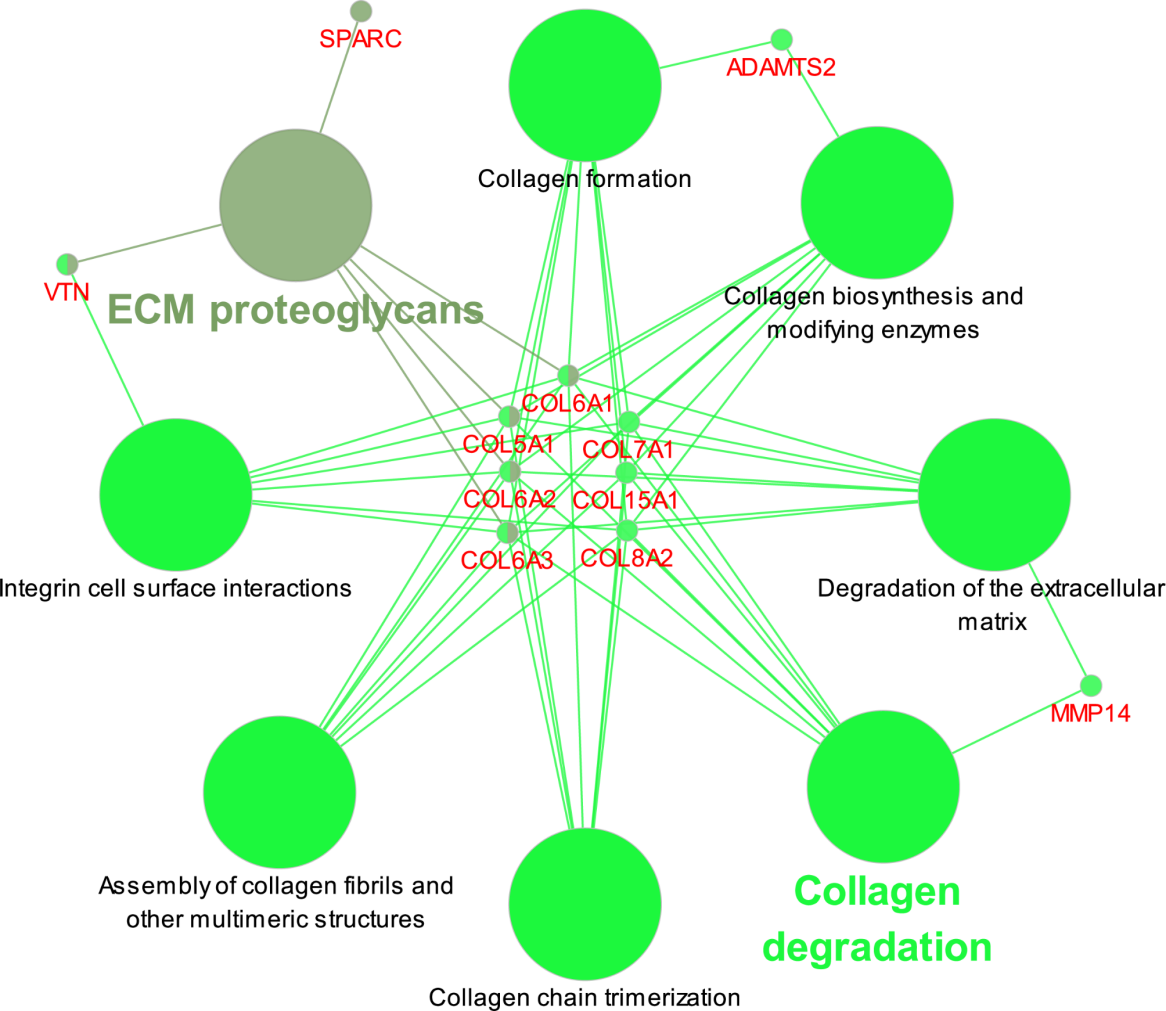
The function enrichment of the 11 hub genes by the ClueGO and CluePedia plugins in Cytoscape

The Reactome function enrichment analysis using the ClueGO and CluePedia plug-in of Cytoscape showed that these hub genes were mainly enriched in collagen degradation and extracellular matrix (ECM) proteoglycans (Fig. 8).

**Table 1:** The m5C-methylated peaks in 11 hub genes

Gene name	Chrom	ThickStart	ThickEnd	Methylation status	Regulation
MMP14	14	22,836,556	22,836,620	Hypermethylated	Upregulated
VTN	17	28,373,011	28,373,091	Hypermethylated	Upregulated
COL15A1	9	98,944,161	98,944,250	Hypermethylated	Upregulated
COL6A2	21	46,098,096	46,098,173	Hypermethylated	Upregulated
SPARC	5	151,686,961	151,687,160	Hypermethylated	Upregulated
COL5A1	9	134,823,415	134,823,469	Hypermethylated	Upregulated
COL6A3	2	237,361,120	237,361,174	Hypermethylated	Upregulated
COL6A1	21	45,998,896	45,998,959	Hypermethylated	Upregulated
COL8A2	1	36,098,541	36,098,760	Hypermethylated	Upregulated
ADAMTS2	5	179,122,643	179,122,740	Hypermethylated	Upregulated
COL7A1	3	48,567,573	48,567,636	Hypermethylated	Upregulated

## Discussion

In the present study, we have given an overview of the distinct m5C modification profiles of mRNAs in OA and normal cartilage for the first time. We found abundant m5C methylation sites on the mRNAs of knee cartilage tissues, especially in degenerative cartilage, indicating an overall upregulated m5C methylation level in OA. This is consistent with previous studies in multiple tumor tissues, which also found rich depositions of m5C in transcriptomes [7, 14, 15]. However, a study by Legrand et al. only found a few m5C methylated mRNAs in the mouse transcriptome, and this suggested that mRNAs were sparsely methylated [16]. Huang et al. collected seven types of human tissue and 11 types of mouse tissue to examine the landscape of m5C in mammals and found a wide range of methylation levels in different tissues [17]. Thus, it can be assumed that the m5C modification levels in mRNAs vary across species and tissues. Our study has indicated that tissue specificity endows OA cartilage with rich m5C site distribution in mRNAs, which is an important basis for m5C to play a critical role in OA pathogenesis. On the other hand, it is still challenging to detect the transcriptome-wide m5C profile accurately and systematically. Several detection methods have been reported for identifying the mRNA m5C map, including MeRIP-Seq, Aza-IP [18], miCLIP [19], and RNA bisulfite sequencing (BS-seq) [20]. These methods have their own benefits and limitations, and no consensus has been reached on the best option. The variable detection results may be partly due to the method selection. These controversial findings prompt the development of more robust techniques for accurate transcriptome-wide m5C detection in mRNAs.

In addition to mRNAs, m5C modification is also widely present in other regulatory non-coding RNAs. Several studies have revealed substantially elevated m5C levels of lncRNAs and circRNAs in carcinoma tissues compared with adjacent non-tumor tissues [15, 21]. In fact, most of the m5C-modified RNAs are ribosomal RNAs (rRNAs) and transfer RNA (tRNAs), and the deposition of m5C in these RNAs imparts important biological functions [22]. For instance, it was found that m5C modification was essential to stabilizing the tRNA secondary structure and maintaining metabolic stability [23]. Changed m5C levels in rRNA could modulate the respective lifespans in yeast, worms and flies [24]. The unmethylated status of 28 S rRNA caused by the loss of an RNA methyltransferase for m5C increased survival under cellular stress [25]. Nevertheless, m5C-related studies on noncoding RNAs in OA pathogenesis are still lacking, and further exploration should be conducted in this area.

KEGG analysis revealed that genes with downregulated m5C peaks were enriched in the PI3K-Akt signaling pathway and the hypoxia-inducible factor-1 (HIF-1) signaling pathway. These two signaling pathways are crucial to OA pathogenesis. Articular cartilage is maintained in a low-oxygen environment in the lifetime of an individual, and HIF-1α is essential for the adaptation of chondrocytes to hypoxia [26]. Thus, HIF-1α is an important protective factor in chondrocyte homeostasis. The PI3K-Akt pathway is also critical in both the normal metabolism of cartilage and OA pathogenesis. Previous studies showed that the PI3K-Akt pathway extensively participates in the inflammatory response, chondrocyte proliferation, apoptosis, and autophagy [27, 28]. The multiple functions of this axis make it difficult to verify the general effects of this signaling pathway in OA, thereby setting up obstacles to the exploration of related therapeutic targets. The dominant effects or the cross-talk among the diverse effects of PI3K-Akt in OA are the prominent issues that need to be addressed. Our study has indicated that m5C modification might participate in the regulation of these signaling pathways in OA and might provide a new perspective for solving the above issues.

We identified 11 m5C-modified hub genes in OA cartilage via bioinformatic analysis, and these hub genes were primarily enriched in collagen degradation and ECM degradation. Progressive ECM degradation is one of the hallmarks of OA. Our results indicate that m5C modification might play an important role in this catabolic process. COL15A1, COL6A2, COL5A1, COL6A3, COL6A1, COL8A2, and COL7A1 are collagen-encoding genes. Multiple studies have shown that these genes are markedly upregulated in OA pathogenesis [29–31]. Type II collagen is a major component of normal articular cartilage, and the elevated expression of ECM turnover-related genes is indicative of ECM component remodeling and aberrant collagen accumulation. It was shown that aberrant collagen-encoding gene expression might contribute to fibrosis [32]. It was also indicated that the ECM turnover-related genes were downstream of the TGF-β signaling pathway in OA-related synovial fibrosis, and OA cartilage damage could stimulate elevated TGF-β expression, thereby facilitating fibrosis [33]. Our results have shown that m5C modification might be involved in the activation of aberrant collagen-encoding genes and suggest another regulatory mechanism of ECM component remodeling in OA cartilage.

MMP14 and ADAMTS2 are well-known metalloproteinases. Their elevated expression considerably reduces some of the dominating components of the ECM in cartilage, such as Type II collagen and aggrecan, thereby promoting OA progression [34]. Due to the important role of metalloproteinases in OA pathogenesis, many metalloproteinase inhibitors have been developed as disease-modifying therapeutics, and some of them have entered clinical trials [35, 36]. Among them, only doxycycline has been approved by the FDA. Nevertheless, the effect of doxycycline on reducing OA symptoms is still controversial. Hsien-Tsung Lu et al. found that injectable hyaluronic acid doxycycline significantly ameliorated the progression of OA [37]. However, a triple-blinded randomized controlled trial showed that doxycycline was ineffective in OA symptom relief in the short and medium term [38]. Further studies on the regulatory mechanism of metalloproteinases in OA pathogenesis are needed for the development of more effective and specific inhibitors. It has been shown that alterations in epigenetic modification can affect the vitality and expression of metalloproteinases. The ubiquitination of MMP14 by FBXO6, a ubiquitin ligase subunit, decreased its expression, leading to inhibited OA development [39]. ADAMTS2 was also recognized as a crucial ECM-related regulator in pancreatic cancer. The knockdown of the m6A demethylase FTO significantly increased the m6A level and decreased the mRNA expression of ADAMTS2 [40]. As the m5C modification effects of these ECM-related protease have never been clarified, we can only make some suggestions based on our findings that the elevated m5C modification of these proteases might contribute to their increased expression and aberrant activity in OA pathogenesis.

It should be acknowledged that our study had several limitations. First, the small sample size may affect the accuracy of the results. Variables other than OA may have an impact on the results, and larger sample sizes can help minimize the bias introduced by these variables. Second, our results were mainly based on high-throughput sequencing and bioinformatics analysis, and further molecular experiments are needed to verify our hypotheses. Despite these defects, we believe our study offers a valuable reference for further investigation of m5C-related therapeutic targets in OA.

## Conclusion

In this study, we provided the first overview of the patterns and characteristics of m5C modifications in OA and normal cartilage tissue. We obtained a rich set of m5C modification sites in mRNA and observed significant differences between the two groups. We believe that our study provides valuable reference for further research on therapeutic targets related to m5C in OA.

## Materials and methods

### Sample collection and ethical approval

Knee cartilage samples of medial condyles were collected from three patients who underwent knee arthroplasty due to advanced OA and three patients who underwent thigh amputation due to trauma. Clinical characteristics of included patients were shown in Table 2. Fresh samples were immediately frozen in liquid nitrogen and stored at -80 ℃ for detection. This study was approved by the institutional ethics board of the First affiliated Hospital of Zhengzhou University.

**Table 2:** Clinical characteristics of included patients

Sample	Age	Gender	BMI	Kellgren-Lawrence Grade (n)
OA 1	67	female	28.5	4
OA 2	66	female	27.4	4
OA 3	72	female	28.8	4
Normal 1	61	female	25.2	0
Normal 2	69	female	29.4	0
Normal 3	68	female	26.6	1

### RNA extraction and preparation

The total RNA from each sample was extracted using TRIzol reagent (Invitrogen Corporation, Carlsbad, CA). The content of rRNA in the total RNA was reduced using the Ribo-Zero rRNA Removal Kit (Illumina, Inc., CA, USA). The concentration of total RNA was measured using a NanoDrop ND-1000 spectrophotometer (NanoDrop Technologies, Wilmington, DE, USA). RNA with an OD260/OD280 ratio range of 1.8–2.1 was marked as acceptable.

### MeRIP-Seq construction and sequencing

The m5C-IP-Seq service was provided by CloudSeq Inc. (Shanghai, China). Total RNA was subjected to immunoprecipitation with the GenSeq® m5C-IP Kit (GenSeq Inc.) by following the manufacturer’s instructions. RNA libraries for IP and input samples were then constructed with the GenSeq® Low Input Whole RNA Library Prep Kit (GenSeq, Inc.) by following the manufacturer’s instructions. Libraries were qualified using the Agilent 2100 bioanalyzer and then sequenced on a NovaSeq platform.

### Sequencing data analysis

Briefly, paired-end reads were harvested from the Illumina Novaseq 6000 sequencer, and Q30 was used for quality control. After 3’ adaptor-trimming and low-quality read removal via cutadapt software (v1.9.3), clean reads of all libraries were aligned to the reference genome (UCSC MM10) using the Hisat2 software (v2.0.4). Methylated sites on RNAs (peaks) were identified using MACS software. Differentially methylated sites were identified by diffReps with a fold change threshold > 2 and P-value < 0.0001. The differentially expressed genes were identified using the DESeq2 R package with a P-value < 0.05 and fold change > 2 as the cutoff criteria. The Gene Ontology (GO) project contains three parts: biological processes (BP), molecular functions (MF), and cellular components (CC). Differentially m5C-modified genes were used to perform GO functional analysis to annotate and speculate on the function of these differentially methylated genes. Pathway analysis using the Kyoto Encyclopedia of Genes and Genomes (KEGG) [41]was conducted with differentially methylated genes for annotation and inference of the pathways they could be involved in. GO and KEGG analyses were performed using the clusterprofile R package (v3.6.0) with a P-value < 0.05 were considered statistically significant.

### Identification and function enrichment analysis of hub genes

The Search Tool for the Retrieval of Interacting Genes/Proteins (STRING) database was used to analyze the PPI network of the selected genes, and Cytoscape (v3.9.1) was used to identify the network modules in the resulting file. The degree, closeness and MNC algorithms of the cytoHubba plugin in Cytoscape (v3.9.1) were used to calculate scores of nodes in the PPI network. The hub genes were determined by overlapping the top 15 genes identified by these algorithms.

The function enrichment analysis of m5C-modified hub genes was performed with the ClueGO [42] and CluePedia [43] plugins of Cytoscape (v.3.9.1). The analysis type is set to REACTOME pathways. Pathways with P < 0.05 were shown.

### Electronic supplementary material

Below is the link to the electronic supplementary material.

Supplementary Material 1

Supplementary Material 2

Supplementary Material 3

Supplementary Material 4

## Data Availability

The data used to support the findings of this study are available in the NCBI repository. The data is accessible via NCBI GEO submission ID: GSE235610. It can be viewed at https://www.ncbi.nlm.nih.gov/geo/query/acc.cgi?acc=GSE235610.
